# Graph neural networks for preference social recommendation

**DOI:** 10.7717/peerj-cs.1393

**Published:** 2023-05-19

**Authors:** Gang-Feng Ma, Xu-Hua Yang, Yue Tong, Yanbo Zhou

**Affiliations:** College of Computer Science and Technology, Zhejiang University of Technology, Hangzhou, Zhejiang, China

**Keywords:** Social recommendation, Social preference network, Graph neural network

## Abstract

Social recommendation aims to improve the performance of recommendation systems with additional social network information. In the state of art, there are two major problems in applying graph neural networks (GNNs) to social recommendation: (i) Social network is connected through social relationships, not item preferences, *i.e.*, there may be connected users with completely different preferences, and (ii) the user representation of current graph neural network layer of social network and user-item interaction network is the output of the mixed user representation of the previous layer, which causes information redundancy. To address the above problems, we propose graph neural networks for preference social recommendation. First, a friend influence indicator is proposed to transform social networks into a new view for describing the similarity of friend preferences. We name the new view the Social Preference Network. Next, we use different GNNs to capture the respective information of the social preference network and the user-item interaction network, which effectively avoids information redundancy. Finally, we use two losses to penalize the unobserved user-item interaction and the unit space vector angle, respectively, to preserve the original connection relationship and widen the distance between positive and negative samples. Experiment results show that the proposed PSR is effective and lightweight for recommendation tasks, especially in dealing with cold-start problems.

## Introduction

Recommendation systems is a hot spot in current network applications and research (Wang, Wang & Yeung, 2015; Ebesu, Shen & Fang, 2018). High-quality recommendations can help users quickly discover interesting content and increase product sales. In recent years, with the rise of graph neural networks, recommendation systems based on graph neural networks, have received extensive attention (Wang et al., 2019). However, the traditional user-item interaction network (U-I network) has the problem of data sparsity (Guo et al., 2019), that will affect the performance of the recommendation system. Social recommendation (Guo, Zhang & Yorke-Smith, 2015) enhances the user representation by introducing additional user-user information, and further enhances the item representation through the information aggregation of the graph neural network. In addition, the recommendation system also suffer from the cold-start problem (Wahab et al., 2022), *i.e.,* the amount of information about the new users is too small for personalized recommendation. Social recommendation assigns an initial preference vector to new users by user-user information. This vector is used to recommend suitable items for new users.

**Figure 1:**
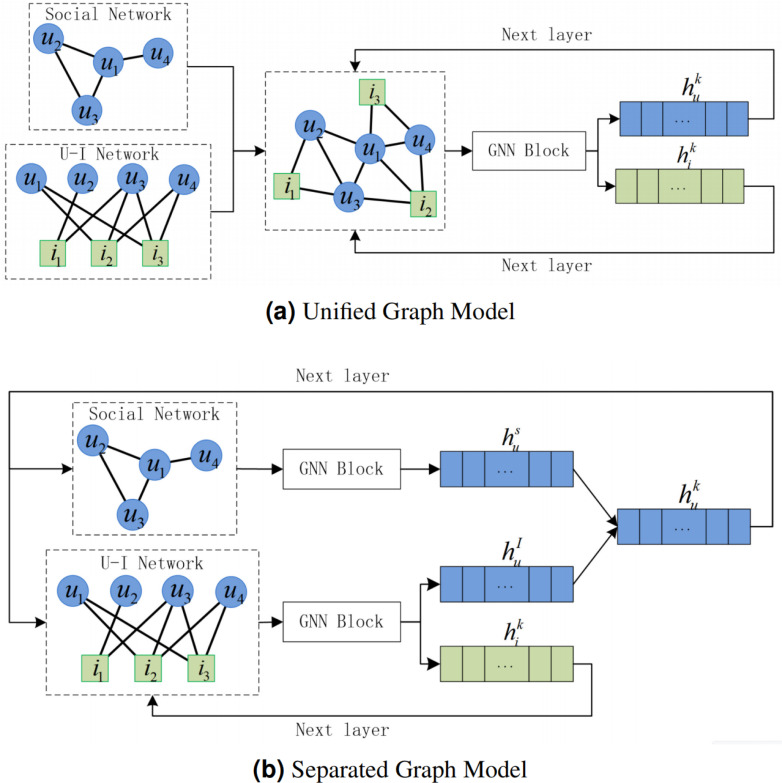
Unified graph model and separated graph model of GNN-based social recommendation, where *u* is user, *i* is item, ${h}_{u}^{S}$ is user representation of social network, ${h}_{u}^{I}$ is user representation of U-I network, ${h}_{u}^{k}$ is the user representation output of layer *k*, ${h}_{i}^{k}$ is the item representation output of layer *k*. (A) Unified graph model, which cannot represent social network and U-I network separately. (B) Separated graph model. ${h}_{u}^{k}$ is the common user input of the two networks of layer *k* + 1. In layer *k* + 1, user representation of social network redundant user-item interaction information, user representation of U-I network redundant user-user interaction information. Aggregating the updated redundant user representations ${h}_{u}^{k+1}$ will cause further redundancy.

In social recommendation, users capture information from social network and U-I network. According to the different integration forms, the social recommendation model based on graph neural network can be divided into unified graph model and separated graph model (Wu et al., 2022). The unified graph model merges the social network and the U-I network, and directly extracts the joint information of the two networks through the graph neural network. As shown in Fig. 1A, in the unified graph, the social network and the U-I network share the same user representation, which effectively ensures the consistency of information updates in both networks. Considering the information differences of users and items, Neural graph collaborative filtering (NGCF) (Wang et al., 2019) designed different aggregation methods for neighboring users and neighboring items. However, the artificial design cannot meet the complex network environment. Diffnet++ (Wu et al., 2020) and SEFrame (Chen & Wong, 2021) used attention mechanism to adaptively capture the information interaction between neighboring users, between neighboring items, and between neighboring users and neighboring items. However, the network is sparse, *i.e.,* there are a large number of unknown connected edge, which leads to the information bias, especially after using attention to highly aggregate the neighbors. Therefore, some social network models used user similarity (Song et al., 2021), generative adversarial networks (Yu et al., 2019), and other methods to complement the network relationships. Both social networks and U-I networks have their own unique information. The unified graph model lacks separate representations of the two networks, which affects the representation performance to some extent. The separated graph model handles the information of social network and U-I network separately, and extracts the information of the two networks through different graph neural networks. Therefore, the choice of graph neural networks is more flexible under the separated graph model. In contrast to the graph neural network-based social recommendation framework (GNN-SoR) (Guo & Wang, 2020) and SocialLGN (Liao et al., 2022) which used a classical graph neural network model, AGREE (Cao et al., 2019) grouped nodes and used attention for each group to capture local information. DANSER (Wu et al., 2019c) further proposed dual attention to capture the interaction between the two graph neural networks. The user representations of social networks and U-I networks obtained from the above separated graph models are able to effectively capture the differences between the two networks. As shown in Fig. 1B, in the separated graph, the user representations of the two networks need to be merged. Diffnet (Wu et al., 2019b) simply summed the two types of user representations to greatly reduce the computational complexity. However, the method needs to ensure that the amount of information contained in the two types of user representations cannot be significantly different. GraphRec (Fan et al., 2019) used a multi-layer neural network to further explore the potential information of the two types of user representations. It can improve the performance of the representations, but may lead to over-fitting. Some articles (Song et al., 2019; Xu et al., 2020; Liao et al., 2022), on the other hand, used the concatenate operation for user presentations, which can solve the difference of the amount of information at a low computational complexity. However, in the existing separated graph model, the user representation output of the current layer is the combination of the user representation of the two networks. Whether it is the social network or the U-I network, it is redundant to use the combined user representation output of the current layer as the input of the next layer of graph neural network. As the number of layers deepens, the redundant information will continue to accumulate.

This article proposes graph neural networks for preference social recommendation (PSR). PSR adopts the separated graph model to fully capture the independent information of social network and U-I network. Compared with the previous separated graph model, we further separate the updating and combining operations of user representations of the two networks to avoid information redundancy. Furthermore, few articles note that not all social network relationships contribute to the U-I network. Social networks are noisy, and friends do not necessarily share the same preferences. Therefore, we propose the social preference network to enhance the social network. The main contributions of the article are summarized as follows.

1. A friend influence indicator is proposed. It captures user preferences through user-item interaction information, and then transforms social networks into social preference networks that are more suitable for recommendation systems.
2. A PSR model is proposed. It can effectively avoid information redundancy, and can fully capture the respective information and joint information of the two networks.
3. Two losses in the objective function are used. These two losses are used to preserve the initial connection relationship between nodes and widen the distance between samples and negative samples, respectively.

The rest of article is organized as follows. Section 2 is related work, Section 3 describe the PSR model, Section 4 is experiment, and Section 5 gives conclusions.

## Related Work

We propose the social preference network. Its main idea is to use heterogeneous networks to complement the heterogeneous information of homogeneous networks, thus reducing the information difference between the two networks. The idea is applicable to social networks and can be generalized to other networks, such as social opportunistic networks (Liu et al., 2018; Zhang et al., 2019). In addition, we use different graph neural network models to capture information from social network and U-I network, respectively. Our approach is based on separated graph model. The following is the related work of the article.

### Graph neural network in social recommendation

Graph neural networks, especially graph convolutional networks, can achieve fast and efficient information aggregation and update through network topology information. GCN (Welling & Kipf, 2016) aggregates neighbor nodes by degree penalty, realizing convolution on the network. NGCF (Wang et al., 2019) changes the GCN convolution kernel by adding additional interaction information between nodes and neighbor nodes, and successfully introduces graph convolution into the recommendation system. LightGCN (He et al., 2020) adopts the idea of SGC (Wu et al., 2019a) which deletes the nonlinear activation function of NGCF. GraphRec (Fan et al., 2019) uses the attention mechanism, and adds rating embedding in aggregation to improve node representation. SEPT (Yu et al., 2021a) refers to the deep graphic infomax (DGI) (Velickovic et al., 2019) model and uses contrastive learning as the loss function to effectively mine the neighborhood information of nodes. Our method uses two modified graph neural network models to update node representations in social networks and U-I networks, respectively.

### Influence of friends in social recommendation

Social recommendation is based on the assumption that the user’s friends will influence the user’s preferences. That is, it is important to explore social relationships in social recommendation. DiffNet (Wu et al., 2019b) believes that the relationship between users and different friends is consistent. This idea is simple, but it may be unrealistic in real situations. GraphRec (Fan et al., 2019), GAT-NSR (Mu et al., 2019) and DGRec (Song et al., 2019) use neural networks to learn the similarity between users and friends, and achieve certain results. DANSER (Wu et al., 2019c) learns the weight of social relation by a dual graph attention to mine the importance of users.

The above separated graph method fully mines the social relationships of users in social networks. However, users’ social relationships are not always positive for item recommendation, that is, friends may have completely different item preferences. To solve this problem, one way is to use the unified graph model. For example, DiffNetLG (Song et al., 2021) uses user similarity to complement social relationships. Since the two networks in the unified graph share the same user representation, the added edge can reflect the item preference relationship between users to a certain extent. However, the unified graph model lacks separate representations for social networks and U-I networks. In separated graph model, a more reasonable method is to mine the influence of friends of users in social networks. The enhanced social recommendation framework (ESRF) (Yu et al., 2020) uses an autoencoder to reconstruct complex and high-order friend influences in networks, and uses the original social network relationship to constrain it to ensure the validity of the obtained user preference relationship network. SEPT (Yu et al., 2021a) mines strongly connected social relationships from the original social network. Then, the social relationships are used to constrain the preference similarity of the original social network. HOSR (Liu et al., 2020) uses topological information to capture high-order social relationships, so as to mine possible consistent item preferences between users who are not directly connected. MHCN (Yu et al., 2021b) uses hypergraphs to model high-order relationships among users, and uses multiple channels to construct different hypergraphs to improve robustness. MTRTrust (Mauro, Ardissono & Hu, 2019) introduces additional user global influence information, which is used to evaluate the importance of different user preferences together with the local influence of users. Our method uses the user’s real item preference to mine the user’s friend influence to ensure the consistency of social relationships and preference relationships. We constrain influence through original social networks to preserve the original social network information.

## Graph Neural Networks for Preference Social Recommendation (PSR)

We propose graph neural networks for preference social recommendation (PSR). The algorithm fully mine the users’ preference and the preference relationship between users.

### Problem description

In this article, we use two network including user-item interaction network (U-I network) *G*_*I*_ = (*U*, *I*, *E*_*I*_) and user-user interaction network (social network) *G*_*S*_ = (*U*, *E*_*S*_), where *U* = {*u*_1_, *u*_2_, …, *u*_*N*_} denotes the user nodes, *I* = {*i*_1_, *i*_2_, …, *i*_*M*_} denotes the item nodes, *E*_*I*_ and *E*_*S*_ represent the edge of the two networks, respectively, and *N* is the number of users, *M* is the number of item. *p*_*u*_ is the user representation of social network, *q*_*u*_ and *q*_*i*_ are the user representation and item representation of U-I network, respectively. *A*_*S*_ ∈ ℝ^*N*×*N*^ is the adjacency matrix of social network and *A*_*I*_ ∈ ℝ^*N*×*M*^ is the rating matrix of U-I network.

Our goal is to enrich the node representation information in U-I network through the generated social preference network.

### Algorithm framework

**Figure 2:**
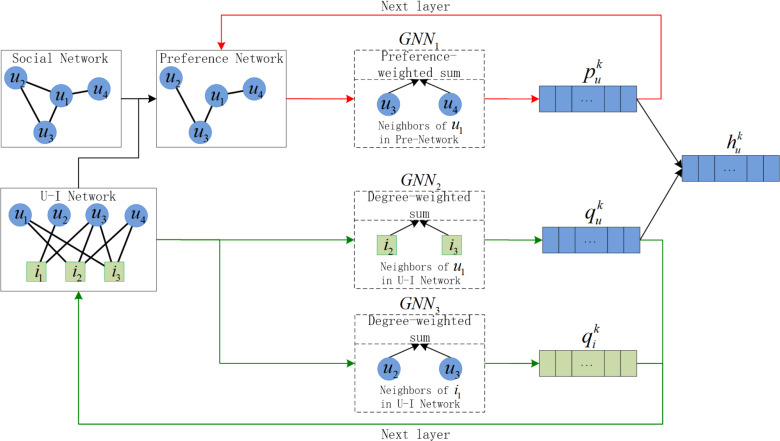
The design of PSR framework, where *u* is user, *i* is item, and ${h}_{u}^{k}$ is the combined user representation of layer *k*. The red line is the update of the node representation of the social preference network, and the green line is the update of the node representation of the U-I network.

As shown in Fig. 2, our algorithm framework consists of three parts:

1. Social preference network representation: The social preference network is constructed by the social network and the U-I network. It uses *GNN*_1_ with *l*-layer parameter sharing for representation, and ${p}_{u}^{k}$ is the user representation output of layer *k*.
2. U-I network representation: U-I network uses *GNN*_2_ with *l*-layer parameter sharing for user representation and uses *GNN*_3_ with *l*-layer parameter sharing for item representation. And ${q}_{u}^{k}$ and ${q}_{i}^{k}$ are corresponding user and item representation output of layer *k*.
3. Two losses-based PSR model training: The final representation is the mean of all layer representations. The two losses are used to preserve the original connection relationship and widen the distance between positive and negative sample.

${h}_{u}^{k}$ is the combined user representation of layer *k*. Instead of ${h}_{u}^{k}$, the algorithm framework chooses ${p}_{u}^{k}$ and ${q}_{u}^{k}$ as the input of next layer of the two network respectively, that well solve the problem of information redundancy.

### Model description

Our model is a separated graph model. For clarity, we disassemble the whole model into the following three parts.

#### Social preference network representation

In most cases, friends will influence each other, resulting in similar preferences, but it is not absolute. If friends have completely different preferences, recommendations based on social relationships are unreliable. This means that social networks cannot necessarily be used directly for recommendation systems, which need to be adjusted beforehand.

We use user preference information of the U-I network to obtain social network friend influence. Analyzing the U-I network, let the item sets of users’ preferences be *H* and the common preference matrix of users be *C*, then the common preference number *C*_*xy*_ between user *x* and user *y* is *C*_*xy*_ = |*H*_*x*_∩*H*_*y*_|. By fully considering the preference relationship and preference difference between users, we propose the friend influence indicator *T*_*xy*_:

(1) \begin{eqnarray*}{T}_{xy}= \frac{{C}_{xy}}{\sqrt{{|}{H}_{x}{|}}\sqrt{{|}{H}_{y}{|}}} .\end{eqnarray*}

We use social network to constrain indicator results to ensure the validity of friend influence. That is, the indicator only calculates the friend influence among connected users in social network. The value range of *T*_*xy*_ is [0, 1]. If *T*_*xy*_ = 0, the mutual influence is 0, which means that there is no common preference between user *x* and user *y*. If *T*_*xy*_ = 1, the mutual influence reaches the maximum, which means that the preference is highly correlated between users. In particular, if all friend influence indicator values *T*_*x*__ of user *x* are all 0, the user *x* only has U-I network information, but no social network information. Taking the friend influence indicator as the edge weight of the social network, and removing edges with weight value of 0, a new view is obtained. In this article, we name the view the Social Preference Network.

Next, we update user representation in the social preference network. We use friend influence as aggregate weight of neighbor nodes, and define the update method of the representation ${p}_{{u}_{x}}^{k}$ of user *x* of layer *k* as

(2) \begin{eqnarray*}{p}_{{u}_{x}}^{k}=\sigma \left( \sum _{y\in {\Gamma }_{x}^{S}\cup x}{T}_{xy}{p}_{{u}_{y}}^{k-1}{W}_{1} \right) \end{eqnarray*}

where ${\Gamma }_{x}^{S}$ is the neighbors of user *x* in social preference network, *σ* is the tanh activation function, *W*_1_ ∈ ℝ^*d*×*d*^ is weight matrix and *d* is the dimension of hidden layer. In particular, *T*_*xy*_ = 1 when *y* = *x*.

We use *D*_*u*_ = *diag*{*D*_*u*_1__, …, *D*_*u*_*N*__} ∈ ℝ^*N*×*N*^ as the diagonal degree matrix, where *D*_*u*_*x*__ = |*H*_*x*_| is the degree of user *x* in U-I network. Combining Eqs. (1) and (2), the matrix formulation of node update of layer *k* in social preference network is expressed as

(3) \begin{eqnarray*}{p}_{u}^{k}=GN{N}_{1}({p}_{u}^{k-1})=\sigma ((C\odot {D}_{u}^{- \frac{1}{2} }{A}_{S}{D}_{u}^{- \frac{1}{2} }+I){p}_{u}^{k-1}{W}_{1})\end{eqnarray*}

where the weight matrix *W*_1_ is shared by parameters in different layers.

#### U-I network representation

The U-I network can well reflect the user’s preference information, that can be directly used in recommendation system. We define the update method of the representation ${q}_{{u}_{x}}^{k}$ of user *x* of layer *k* as

(4) \begin{eqnarray*}{q}_{{u}_{x}}^{k}=\sigma (\sum _{y\in {\Gamma }_{x}^{I}} \frac{1}{\sqrt{{D}_{{u}_{x}}{D}_{{i}_{y}}}} {q}_{{i}_{y}}^{k-1}{W}_{2})\end{eqnarray*}

where ${\Gamma }_{x}^{I}$ is the neighbor item set of user *x*, and *D*_*u*_*x*__ and *D*_*i*_*y*__ represent the degree of user *x* and item *y* respectively in U-I network. In order to be consistent with the user representation of social preference network, we use the same activation function *σ* = *tanh* and the same dimension of the weight matrix *W*_2_ ∈ ℝ^*d*×*d*^.

In order to reduce the number of parameters and the computational complexity, we delete the weight matrix and nonlinear activation function used in item update. Therefore, we define the update method of the representation ${q}_{{i}_{z}}^{k}$ of item *z* of layer *k* as

(5) \begin{eqnarray*}{q}_{{i}_{z}}^{k}=\sum _{y\in {\Gamma }_{z}^{I}} \frac{1}{\sqrt{{D}_{{i}_{z}}{D}_{{u}_{y}}}} {q}_{{u}_{y}}^{k-1}\end{eqnarray*}

where ${\Gamma }_{z}^{I}$ is neighbor user set of item *z*, and *D*_*i*_*z*__ and *D*_*u*_*y*__ represent the degree of item *z* and user *y* respectively in U-I network.

We use *D* = *diag*{*D*_*u*_1__, …, *D*_*u*_*N*__, *D*_*i*_1__, …, *D*_*i*_*M*__} ∈ ℝ^(*N*+*M*)×(*N*+*M*)^ as the diagonal degree matrix. The corresponding adjacency matrix *A* ∈ ℝ^(*N*+*M*)×(*N*+*M*)^ is

(6) \begin{eqnarray*} \left( \begin{array}{@{}cc@{}} \displaystyle \mathbf{0}&\displaystyle {A}_{I}\\ \displaystyle {A}_{I}^{T}&\displaystyle \mathbf{0}. \end{array} \right) \end{eqnarray*}

Combining Eqs. (4), (5) and (6), the matrix formulation of node update of layer *k* in U-I network is expressed as

(7) \begin{eqnarray*} \left( \begin{array}{@{}c@{}} \displaystyle {q}_{u}^{k}\\ \displaystyle {q}_{i}^{k} \end{array} \right) = \left( \begin{array}{@{}c@{}} \displaystyle GN{N}_{2}({q}_{u}^{k-1})\\ \displaystyle GN{N}_{3}({q}_{i}^{k-1}) \end{array} \right) =f({D}^{- \frac{1}{2} }A{D}^{- \frac{1}{2} } \left( \begin{array}{@{}c@{}} \displaystyle {q}_{u}^{k-1}{W}_{2}\\ \displaystyle {q}_{i}^{k-1} \end{array} \right) )\end{eqnarray*}

where *f* is activation function, the first *N* lines *f*(*x*) = *σ*(*x*), the last *M* lines *f*(*x*) = *x*, and the weight matrix *W*_2_ is shared by parameters in different layers.

#### Two losses-based PSR model training

We use a full connected layer to process the user representation of the social preference network and the U-I network to obtain the final user representation *h*_*u*_

(8) \begin{eqnarray*}{h}_{u}= \frac{1}{l} \sum _{k=1}^{l}({p}_{u}^{k}{|}{|}{q}_{u}^{k}){W}_{3}\end{eqnarray*}

where || means concatenate, *W*_3_ ∈ ℝ^2*d*×*d*^ is weight matrix. The final item representation *h*_*i*_ is

(9) \begin{eqnarray*}{h}_{i}= \frac{1}{l} \sum _{k=1}^{l}{q}_{i}^{k}.\end{eqnarray*}

We use inner product *y*_*xz*_ = *h*_*u*_*x*__⋅*h*_*i*_*z*__ as the rating of user *x* and item *z*. By minimizing Bayesian Personalized Ranking (BPR) loss (Rendle et al., 2009) and Global Orthogonal Regularization (GOR) loss (Zhang et al., 2017), the model is trained. BPR loss is used to preserve the original connection relationship, the calculation formula is

(10) \begin{eqnarray*}{L}_{BPR}=\sum _{(x,z)\in {E}_{I}\cup (x,{z}^{{^{\prime}}})\not \in {E}_{I}}-ln(\sigma ({y}_{xz}-{y}_{x{z}^{{^{\prime}}}}))+\lambda {|}{|}{E}^{0}{|}{\mathop{{|}\nolimits }\nolimits }_{2}^{2}\end{eqnarray*}

where ${E}^{0}=[{h}_{u}^{0}{|}{|}{q}_{i}^{0}]$, ${h}_{u}^{0}={p}_{u}^{0}={q}_{u}^{0}$ is randomly initialized user representation, ${q}_{i}^{0}$ is randomly initialized item representation. To keep consistent with the comparison algorithm (Liao et al., 2022), *λ* is set to 1*e*^−4^.

GOR loss is used to widen the distance between positive and negative samples, and the calculation formula is

(11) \begin{eqnarray*}{L}_{GOR}=( \frac{1}{{N}^{{^{\prime}}}} \sum _{(x,{z}^{{^{\prime}}})\not \in {E}_{I}}{y}_{x{z}^{{^{\prime}}}})^{2}+\phi ( \frac{1}{{N}^{{^{\prime}}}} \sum _{(x,{z}^{{^{\prime}}})\not \in {E}_{I}}{y}_{x{z}^{{^{\prime}}}}^{2}- \frac{1}{d} )\end{eqnarray*}

where *N*′ is the number of negative samples. To make the activation function *ϕ* smoother, we use *Softplus* instead of *RELU* of the original article.

Through Eqs. (10) and (11), the final loss function is obtained as

(12) \begin{eqnarray*}L={L}_{BPR}+\alpha {L}_{GOR}\end{eqnarray*}

where *α* is hyperparameter used to balance the two loss.

### Model implementation steps

We propose graph neural networks for preference social recommendation (PSR). It proposes a social preference network to transform the friend relationship in social network into the item preference relationship. In addition, PSR proposes a social recommendation model that can effectively reduce information redundancy, and uses two losses to constrain the obtained node representation. The specific implementation process of the model is shown in algorithm 1.


 
_______________________ 
 Algorithm 1: The running process of PSR                                   _________ 
    Input  : Social network GS, U-I network GI, the number of neural 
                 network layer l 
    Output: The final user representation hu, the final item representation 
                 hi 
  1  Construct the social preference network with Equation 1; 
  2  for each iteraction do 
     3   for k = 1,2,...,l do 
    4   Obtain user presentation pku of layer k in social preference 
network with Equation 3; 
5   Obtain user presentation qku and item presentation qki of layer k 
in U-I network with Equation 7; 
6   The final user representation hu = 1 
l ∑l 
       k=1(pk 
u||qk 
u)W3, where W3 is 
    the weight matrix; 
7   The final item representation hi = 1 
l ∑l 
       k=1 qk 
i ; 
8   Jointly optimize the overall objective in Equation 12    


### Computational complexity

We analyze the space and time complexity of PSR, and add LightGCN (He et al., 2020) and SocialLGN (Liao et al., 2022) for comparison.

#### Space complexity

In PSR, there are two parts of trainable parameters: (i) initial representation of the node, and (ii) weight matrix of neural network. For (i), the space complexity is (*N* + *M*)*d*, which is consistent with most neural network models (*e.g.*, LightGCN and SocialLGN). For (ii), PSR uses three weight matrixes *W*_1_ ∈ ℝ^*d*×*d*^, *W*_2_ ∈ ℝ^*d*×*d*^ and *W*_3_ ∈ ℝ^2*d*×*d*^. Since each weight matrix is parameter-shared among layers, the space complexity of PSR in this part is 4*d*^2^. In summary, the total space complexity of PSR is (*N* + *M* + 4*d*)*d*, which is consistent with SocialLGN. Since *min*(*N*, *M*) ≫ *d*, 4*d* can be ignored, which means that the space complexity of PSR is also approximately equal to LightGCN.

#### Time complexity

Similar to most graph convolution kernels, the friend influence indicator can be calculated as preprocessing. For a single-layer neural network, considering the sparsity of the network, the time complexity of node aggregation of social network is $\mathcal{O}({|}{E}_{S}{|}d)$, while U-I network is $\mathcal{O}({|}{E}_{I}{|}d)$, and the time complexity of the graph diffusion operation through weight matrix is $\mathcal{O}(4N{d}^{2})$. Therefore, the total time complexity of PSR is $\mathcal{O}({|}{E}_{S}{|}dl+{|}{E}_{I}{|}dl+4N{d}^{2}l)$ which is linearly related to *max*(|*E*_*S*_|, |*E*_*I*_|, *N*). It is consistent with SocialLGN, which means that it is lower than most existing GNN-based social recommendation models (SocialLGN is a light GNN-based model).

## Experiments

### Experimental settings

#### Datasets

We use LastFM (Yu et al., 2021a; Yu et al., 2021b) and Ciao (Fan et al., 2019; Fan et al., 2020) to analyze the performance of model. These two datasets are real-world datasets that are often used in recommendation systems. As a music dataset, LastFM includes friend relationships and users’ music preferences. As an online shopping dataset, Ciao includes friend relationships and users’ shopping information. The dataset statistics are shown in Table 1.

#### Comparison algorithms

We compare PSR with some well-known methods to verify the performance of the model. The comparison algorithms are as follows.

- **BPR** (Rendle et al., 2009): A non-graph neural network model which ranks items by maximizing the posterior probability.

- **SBPR** (Zhao, McAuley & King, 2014): The first model to introduce social relationships into recommender systems, that sorts the items according to the user’s preference, the user’s friend’s preference, and the remaining preference.

- **DiffNet** (Wu et al., 2019b): It treats friends influence equally, and updates user and item information by accumulation.

- **NGCF** (Wang et al., 2019): A social recommendation model which only aggregates neighborhood information without aggregating central node information.

- **LightGCN** (He et al., 2020): It removes the weight matrix and nonlinear activation function in NGCF.

- **SocialLGN** (Liao et al., 2022): It designs a graph fusion component for user update, and removes the nonlinear activation function and weight matrix for item update.

**Table 1:** Statistics of the datastes.

Dataset	LastFM	Ciao
# of Users	1,892	7,375
# of Items	17,632	105,114
# of *E*_*I*_	92,834	284,086
# of Density (*E*_*I*_)	0.278%	0.037%
# of *E*_*S*_	25.434	57.544
# of Density (*E*_*S*_)	0.711%	0.106%

#### Parameter settings

For better comparison, we keep aligned with the experimental settings of the current SOTA model (SocialLGN). We take 80% of the data as the training set. And we set the random seed to 2020, the representation dimension to 64, the number of neural network layers *l* to 3, *λ* to 1*e*^−4^, the initial learning rate to 1*e*^−3^ and Adam as the optimizer. For the new hyperparameter *α* in PSR, we choose from {0, 1, …, 10}. In order to reduce the influence of hyperparameters *α*, we choose *α* = 5 by default.

#### Evaluation indicators

We use three mainstream evaluation indicators (Wu et al., 2020; Liao et al., 2022), namely Precision@K, Recall@K, and NDCG@K, to evaluate the recommendation performance of top-K ranking.

Precision@K indicates the probability of correct prediction in the predicted positive sample set.

(13) \begin{eqnarray*}Precision@K= \frac{TP@K}{TP@K+FP@K} \end{eqnarray*}

Recall@K indicates the probability of correct prediction in the real positive sample set.

(14) \begin{eqnarray*}Recall@K= \frac{TP@K}{TP@K+FN@K} \end{eqnarray*}

For the connection relationship of the U-I network, *TP* is the number of predicted connected edges which are actually connected, *FP* is the number of predicted connected edges which are actually disconnected, *FN* is the number of predicted disconnected edges which are actually connected.

NDCG@K considers the ranking order of the prediction results on the basis of the above two indicators, and the formula is

(15) \begin{eqnarray*}NDCG@K= \frac{\sum _{i=1}^{K} \frac{re{l}_{i}}{{\log \nolimits }_{2}(i+1)} }{\sum _{i=1}^{{|}REL{|}} \frac{re{l}_{i}}{{\log \nolimits }_{2}(i+1)} } \end{eqnarray*}

where *rel*_*i*_ is relevance score, |*REL*| is the ranking result under the similarity.

### Recommendation performance evaluation

PSR is compared with 6 well-known algorithms under the LastFM and Ciao datasets. We use Precision@K, Recall@K, NDCG@K to evaluate the recommendation performance, where the value of *K* is {10, 20}. The results are shown in Table 2. In recommendation systems, considering the importance of the cold-start problem, we also do the experiment under cold start. Cold start refers to personalized recommendation for new users. Table 3 is the experimental results under cold start.

Tables 2 and 3 show that PSR obtains the best results for 11/12 indicators in LastFM dataset. In particular, in the cold start experiment, the PSR increases by an average of 21.8% compared to SocialLGN. In the Ciao dataset, PSR obtains the best results for 9/12 indicators. From Table 1, it can be seen that the social network density of the Ciao dataset is low, which means that there may be a lot of missing social information. For PSR model, the generation of the social preference network is constrained by the social network, therefore, the performance improvement of the model under the Ciao dataset is lower than that of the LastFM dataset. Next, we further analyze the evaluation indicators. Under the Precision index, PSR obtains the optimal performance, which indicates that PSR has the highest prediction accuracy for user preferences. Under the Recall index, PSR obtains 6/8 optimal performance, which indicates that PSR has the highest real accuracy for user preferences overall. Similarly, under the NDCG index, PSR achieves 6/8 optimal performance, which shows that PSR is able to rank the importance of the preferred items well. When *k* = 10, PSR obtains 9/12 optimal results, while when *k* = 20, PSR obtains 11/12 optimal results. This shows that PSR is relatively more suitable for the recommendation of multiple number of items.

### Parameter sensitivity analysis

**Figure 3:**
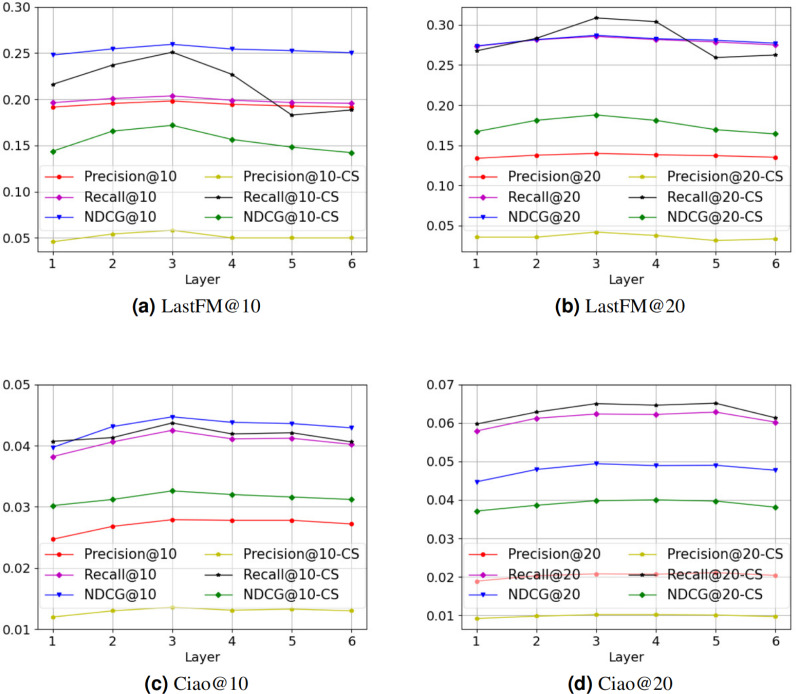
(A–D) The parameter sensitivity experiment of *l*.

**Figure 4:**
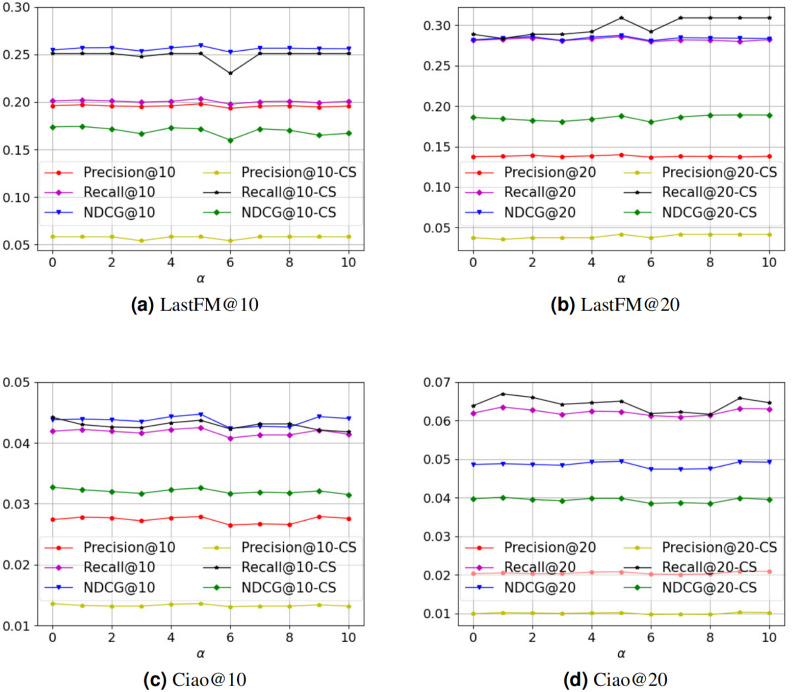
(A–D) The parameter sensitivity experiment of *α*.

There are two core hyperparameters in PSR: (i) the number of neural network layers *l*, and (ii) the value of hyperparameter *α*. We conduct experiments on these two hyperparameters to analyze the parameter sensitivity of PSR. Figure 3 is the parameter sensitivity experiment of *l*, and Fig. 4 is the parameter sensitivity experiment of *α*, where CS means cold start.

**Table 2:** General recommendation performance comparison.

Dataset	Metrics	BPR	SBPR	DiffNet	NGCF	LightGCN	SocialLGN	PSR	Improv.
LastFM	Precision@10	0.0922	0.1398	0.1727	0.1766	0.1961	0.1972	**0.1981**	0.4564%
	Precision@20	0.0720	0.1010	0.1215	0.1269	0.1358	0.1368	**0.1398**	2.1930%
	Recall@10	0.0962	0.1442	0.1779	0.1796	0.2003	0.2026	**0.2036**	0.4936%
	Recall@20	0.1499	0.2070	0.2488	0.2576	0.2769	0.2794	**0.2857**	2.2548%
	NDCG@10	0.1099	0.1749	0.2219	0.2287	0.2536	0.2566	**0.2594**	1.0912%
	NDCG@20	0.1321	0.1978	0.2472	0.2563	0.2788	**0.2883**	0.2871	−0.4162%
Ciao	Precision@10	0.0145	0.0179	0.0238	0.0228	0.0271	0.0276	**0.0279**	1.0870%
	Precision@20	0.0111	0.0141	0.0182	0.0179	0.0202	0.0205	**0.0208**	1.4634%
	Recall@10	0.0220	0.0259	0.0341	0.0343	0.0410	**0.0430**	0.0425	−1.1628%
	Recall@20	0.0339	0.0412	0.0527	0.0531	0.0591	0.0618	**0.0623**	0.8091%
	NDCG@10	0.0229	0.0266	0.0359	0.0359	0.0437	0.0441	**0.0447**	1.3605%
	NDCG@20	0.0260	0.0307	0.0403	0.0407	0.0478	0.0486	**0.0494**	1.6461%

**Notes.**

The best results are highlighted in bold in each row.

**Table 3:** Cold-start recommendation performance comparison.

Dataset	Metrics	BPR	SBPR	DiffNet	NGCF	LightGCN	SocialLGN	PSR	Improv.
LastFM	Precision@10	0.0282	0.0292	0.0417	0.0333	0.0417	0.0458	**0.0583**	27.2926%
	Precision@20	0.0209	0.0333	0.0271	0.0292	0.0313	0.0333	**0.0417**	25.2252%
	Recall@10	0.1151	0.1123	0.1713	0.1169	0.1727	0.1974	**0.2509**	27.1023%
	Recall@20	0.1615	0.2467	0.2407	0.2141	0.2416	0.2663	**0.3087**	15.9219%
	NDCG@10	0.0828	0.0709	0.1107	0.1074	0.1374	0.1419	**0.1718**	21.0712%
	NDCG@20	0.0989	0.1159	0.1309	0.1411	0.1560	0.1643	**0.1878**	14.3031%
Ciao	Precision@10	0.0061	0.0070	0.0104	0.0104	0.0131	0.0134	**0.0136**	1.4925%
	Precision@20	0.0047	0.0060	0.0081	0.0085	0.0096	0.0097	**0.0102**	5.1546%
	Recall@10	0.0208	0.0234	0.0339	0.0341	0.0429	**0.0441**	0.0437	−0.9070%
	Recall@20	0.0328	0.0384	0.0539	0.0557	0.0616	0.0630	**0.0650**	3.1746%
	NDCG@10	0.0138	0.0165	0.0248	0.0245	0.0319	**0.0328**	0.0326	−0.6098%
	NDCG@20	0.0179	0.0219	0.0316	0.0319	0.0384	0.0394	**0.0398**	1.0152%

**Notes.**

The best results are highlighted in bold in each row.

From Fig. 3, with the increase of the number of neural network layers, it can be seen that the overall performance of PSR tends to rise first and then decline. When *l* = 3, PSR achieves the best overall performance. When *l* > 3, PSR suffers from oversmoothing (Welling & Kipf, 2016), which leads to performance degradation. Compared to the Ciao dataset, the performance of the LastFM dataset is more variable. We think the possible reason is that the LastFM dataset is composed of multiple disconnected sub-networks. The different sub-networks have different structures, resulting in different speeds of oversmoothing. In contrast, the network composed of the Ciao dataset is a connected graph, making the speed of oversmoothing relatively consistent. Therefore, the LastFM dataset is more variable in the over-smoothing problem. Compared with the general recommendation performance, the recommendation performance of cold-start is more affected by the oversmoothing. We think the possible reason is that the cold-start user representations rely entirely on the connected user representations in social network, which are more sensitive. From Fig. 4, it can be seen that PSR is less sensitive to hyperparameter *α*. In Section 4.2, in order to obtain the best overall performance, we finally choose *α* = 5.

### Ablation experiment

We analyze each part of the PSR through ablation experiments. Table 4 shows the performance of the PSR model under different ablation experiments, where PSR-BPR is the model without BPR loss, PSR-GOR is the model without GOR loss, PSR-Pre is the model without social preference network, PSR-each is the model that replaces respective user representation with redundant combined user representation as the next layer input, PSR-item is the model that adds weight matrix and tanh activation function to item, PSR-cat is the model that changes the concatenate operation in Eq. (8) to addition, and PSR-output is the model that only uses the output layer as node representation.

PSR obtains 11/12 optimal results, proving the necessity of each component of PSR. Similar to the analysis in Section 4.2, we believe that the Ciao dataset lacks a lot of social information, that reduces the information gains by transforming the social network into the social preference network. Therefore, PSR-Pre achieves two optimal results in the Ciao dataset. The experimental results of PSR-BPR and PSR-GOR demonstrate the necessity of GOR loss and BPR loss. The experimental results of PSR-each show that redundant user representations used by previous articles based on separated graph models would damage the final recommendation performance. PSR-output uses the representation of the last layer, and PSR uses the average of the representations over different layers. Both can capture different order information of nodes. However, PSR is able to capture richer information about the network structure compared to PSR-output. Moreover, the average operation of PSR is equivalent to reducing the weight of high-order information and increasing the weight of low-order information. This implies a hypothesis that the closer the information is to the node, the more important it is to the node. In addition, the average operation, to some extent, can alleviate the possible oversmoothing problem of the last layer representation. And, the experimental results also show that the recommendation performance of PSR-output is lower than that of PSR. Compared with the addition operation used by PSR-cat, the concatenate operation used by PSR is better. It indicates that a great information difference exists between user representations in social networks and U-I networks. Compared with PSR-item, PSR has less number of parameters and computational complexity, but obtains better performance. We try to analyze the possible reasons for this. PSR-item is the model that adds weight matrix and a nonlinear activation function to items in U-I network. Compared with PSR, PSR-item can better fit the relationship between user representations and item representations in U-I networks and thus improve the recommendation performance. However, in social recommendation, we fit the relationship between user representations with additional social network information and item representations. Therefore, the item representations which over-fit U-I network information may affect the final social recommendation performance.

**Table 4:** Ablation experiment.

Dataset	Metrics	PSR	PSR-BPR	PSR-GOR	PSR-Pre	PSR-each	PSR-item	PSR-cat	PSR-output
LastFM	Precision@10	**0.1981**	0.1885	0.1961	0.1962	0.1956	0.1910	0.1931	0.0667
	Precision@20	**0.1398**	0.1322	0.1376	0.1388	0.1362	0.1351	0.1369	0.0509
	Recall@10	**0.2036**	0.1922	0.2011	0.2007	0.2010	0.1948	0.1974	0.0690
	Recall@20	**0.2857**	0.2700	0.2810	0.2832	0.2784	0,2754	0.2794	0.1044
	NDCG@10	**0.2594**	0.2421	0.2546	0.2576	0.2543	0.2440	0.2524	0.0886
	NDCG@20	**0.2871**	0.2689	0.2816	0.2851	0.2805	0.2723	0.2810	0.1027
Ciao	Precision@10	**0.0279**	0.0225	0.0274	**0.0279**	0.0263	0.0238	0.0238	0.0186
	Precision@20	**0.0208**	0.0173	0.0203	0.0207	0.0198	0.0188	0.0188	0.0134
	Recall@10	**0.0425**	0.0345	0.0419	0.0413	0.0392	0.0367	0.0367	0.0262
	Recall@20	0.0623	0.0532	0.0619	**0.0628**	0.0596	0.0582	0.0582	0.0379
	NDCG@10	**0.0447**	0.0358	0.0438	0.0439	0.0416	0.0357	0.0357	0.0289
	NDCG@20	**0.0494**	0.0406	0.0486	0.0491	0.0467	0.0419	0.0419	0.0313

**Notes.**

The best results are highlighted in bold in each row.

## Conclusion

In this article, we propose an approach called graph neural networks for preference social recommendation (PSR). The approach proposes the social preference network, which is used to solve the problem of inconsistency between friend relations and preference relations. Next, PSR uses a separated graph model. By independently updating the social network and U-I network, it reduces information redundancy and fully captures the information of each of networks. Finally, PSR uses two losses to preserve the original connection relationship and widen the distance between positive and negative samples, respectively. Experimental results show that PSR has good performance in social recommendation, especially in cold start. Our approach provides an initial exploration of preference relations in social networks, which may be affected by the sparsity of social network. In the future, we will focus on the social network with large amount of missing information, and further mine user’s preference relationships to generate a more suitable social preference network, so as to improve the performance of social recommendation.

## Additional Information and Declarations

### Data Availability

The following information was supplied regarding data availability: The LastFM dataset was built by Ignacio Fernandez-Tobias with the collaboration of Ivan Cantador and Alejandro Bellogin, members of the Information Retrieval group at Universidad Autonoma de Madrid (http://ir.ii.uam.es):
https://grouplens.org/datasets/hetrec-2011/. Cantador I, Brusilovsky P, Kuflik T. (2011) Second workshop on information heterogeneity and fusion in recommender systems (HetRec2011). In Proceedings of the fifth ACM conference on Recommender systems, pages 387-388. https://doi.org/10.1145/2043932.2044016. The Ciao dataset was built by Jiliang Tang, members of the Data Science and Engineering Lab at Michigan State University:
https://www.cse.msu.edu/ tangjili/datasetcode/truststudy.htm
Tang J, Gao H, Liu H. (2012) mTrust: Discerning multi-faceted trust in a connected world. In Proceedings of the fifth ACM international conference on Web search and data mining, pages 93-102. https://doi.org/10.1145/2124295.2124309
The code is available at Zenodo: Andrewsama. (2023). Andrewsama/PSR: First release of PSR (v1.0.0). Zenodo. https://doi.org/10.5281/zenodo.7769024
